# Chondrocyte‐like nested cells in the aged intervertebral disc are late‐stage nucleus pulposus cells

**DOI:** 10.1111/acel.13006

**Published:** 2019-07-10

**Authors:** Sarthak Mohanty, Robert Pinelli, Paul Pricop, Todd J. Albert, Chitra Lekha Dahia

**Affiliations:** ^1^ Hospital for Special Surgery New York NY USA; ^2^ Weill Cornell Medical College New York NY USA; ^3^ Department of Cell and Developmental Biology, Weill Cornell Medicine Graduate School of Medical Sciences New York NY USA

**Keywords:** Brachyury, chondrocyte‐like cells, fate mapping, Krt19, nucleus pulposus, Shh, trans‐differentiation

## Abstract

Aging is a major risk factor of intervertebral disc degeneration and a leading cause of back pain. Pathological changes associated with disc degeneration include the absence of large, vacuolated and reticular‐shaped nucleus pulposus cells, and appearance of smaller cells nested in lacunae. These small nested cells are conventionally described as chondrocyte‐like cells; however, their origin in the intervertebral disc is unknown. Here, using a genetic mouse model and a fate mapping strategy, we have found that the chondrocyte‐like cells in degenerating intervertebral discs are, in fact, nucleus pulposus cells. With aging, the nucleus pulposus cells fuse their cell membranes to form the nested lacunae. Next, we characterized the expression of sonic hedgehog (SHH), crucial for the maintenance of nucleus pulposus cells, and found that as intervertebral discs age and degenerate, expression of SHH and its target Brachyury is gradually lost. The results indicate that the chondrocyte‐like phenotype represents a terminal stage of differentiation preceding loss of nucleus pulposus cells and disc collapse.

## 1 INTRODUCTION/RESULTS/DISCUSSION

**Figure 1:**
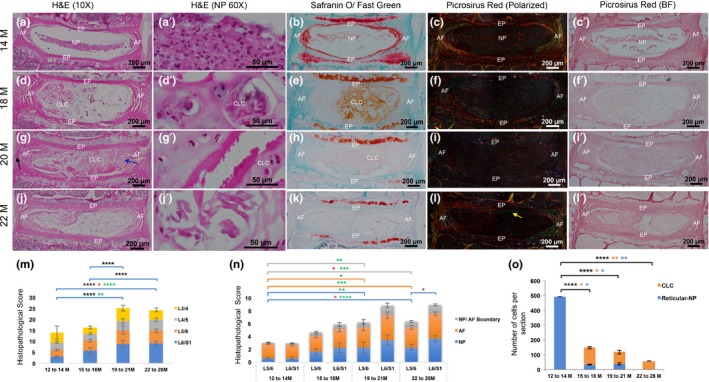
Histopathological changes in the mouse lumbar discs with age. Mid‐coronal sections of 14M (a–c’), 18M (d–f’), 20M (g–i’) and 22M (j–l’)‐old mouse discs stained for H&E (a, a’, d, d’, g, g’, j and j’), SafO/Fast green (b, e, h and k) and picrosirius red (c, c’, f, f’, i, i’, l and l’). m and n, histopathological scoring of 14M to 28M old mouse lumbar discs. o, proportions of reticular NP and CLC with age. M, month. m and n: black *, significance in total score. Red *, significance in L5‐L6 pathology. Green *, significance in L6‐S1 pathology. Purple *, significance between L5‐L6 and L6‐S1. n: Blue line, NP pathology. Orange line, AF pathology. Grey line, NP/AF boundary. o: black *, significance in total number of NP cells. Orange *, significant increase in proportion of CLC‐NP cells. Blue *, significant reduction in proportion of reticular NP cells. *<.05, **<.01, ***<.001 ****<.0001. One‐way ANOVA with post hoc Tukey's test

Degenerative disc disease is a leading cause of low back pain and is associated with structural failure of the intervertebral discs (IVDs or disc). Each disc has a central core of proteoglycan‐rich nucleus pulposus (NP) derived from the embryonic notochord (Choi, Cohn, & Harfe, 2008). NP is surrounded by orthogonal layers of collagenous annulus fibrosus (AF). Both NP and AF are sandwiched between cartilaginous endplates (EP) (Figure 1a). The pathological changes associated with disc aging include hypocellularity, low extracellular matrix (ECM) and reduced disc height (Johnson, Caterson, Eisenstein, & Roberts, 2005; Kauppila, 1995). Despite its prevalence, the causes of age‐related disc pathology are largely unknown. Notochord cells are large and described as “physaliphorous” due to their large vacuoles (Trout, Buckwalter, Moore, Buckwalter, Moore, & Landas, 1982), which is used as a criterion to distinguish notochord cells from non‐notochordal cells in the disc (Hunter, Matyas, & Duncan, 2004). The disappearance of large notochord cells and appearance of smaller clusters of cells, described as “chondrocyte‐like cells” (CLCs), are indicative of disc pathology (Hunter et al., 2004; Trout, Buckwalter, & Moore, 1982). CLCs are nested in a dense, pericellular, osmophilic matrix of unknown composition (Trout et al., 1982). It remains controversial whether vacuolated notochordal cells, mature NP cells and CLCs comprise a single lineage of differentiated phenotypes (Choi, Johnson, & Risbud, 2015; Mwale, 2013), or whether the CLCs represent invasion of surrounding cells (Kim et al., 2003). Establishing the identity of CLCs, and how they appear in degenerated discs, will help design therapeutics targeted towards them. Hence, in this study, using a genetic mouse model we lineage‐traced NP cells to determine whether they differentiate into CLCs with age.

Previous studies showed appearance of CLCs between one and two years of age in mouse lumbar discs (Winkler, Mahoney, Sinner, Wylie, & Dahia, 2014). Here, we timestamped the appearance of CLCs in mouse lumbar discs between 14 and 28 months (M) of age (Figure 1 and Table S1). At 14M, the NP cells were reticular but compact; the AF was organized in thin layers; and a clear NP/AF boundary was observed (Figure 1a–c’,m and n). By 18M, nested CLCs were observed, and the AF layers were disorganized (Figure 1d–f, m and n). By 20M, the disc was hypocellular with outer AF bulging outwards (black arrow), and the inner AF moving into the NP space (blue arrow, Figure 1g–i’). By 22M, the centre of disc was largely acellular (Figure 1j–l’ and o). The NP/AF boundary was lost (Figure 1n). SafO/Fast green staining showed a decrease in the proteoglycan content with age (Figure 1b, e, h and k). Polarized imaging of the picrosirius red‐stained sections shows a decline in thick red and orange collagen fibrils (Figure 1c, f, i and l) while thin green collagen fibrils from the inner EP invaded the NP space (yellow arrow, Figure 1l). Histopathological scoring indicates that the L5‐S1 discs are the most affected (Figure 1m and n, Tables S2 and S3) and the disc content changed from reticular NP to CLC with aging (Figures 1o and 1 and Table S4). These age‐related changes are similar to those reported in human lumbar discs (Trout et al., 1982; Weiler et al., 2010). As CLCs were seen at 16 to 18M of age, we chose this time point for lineage tracing studies.

**Figure 2:**
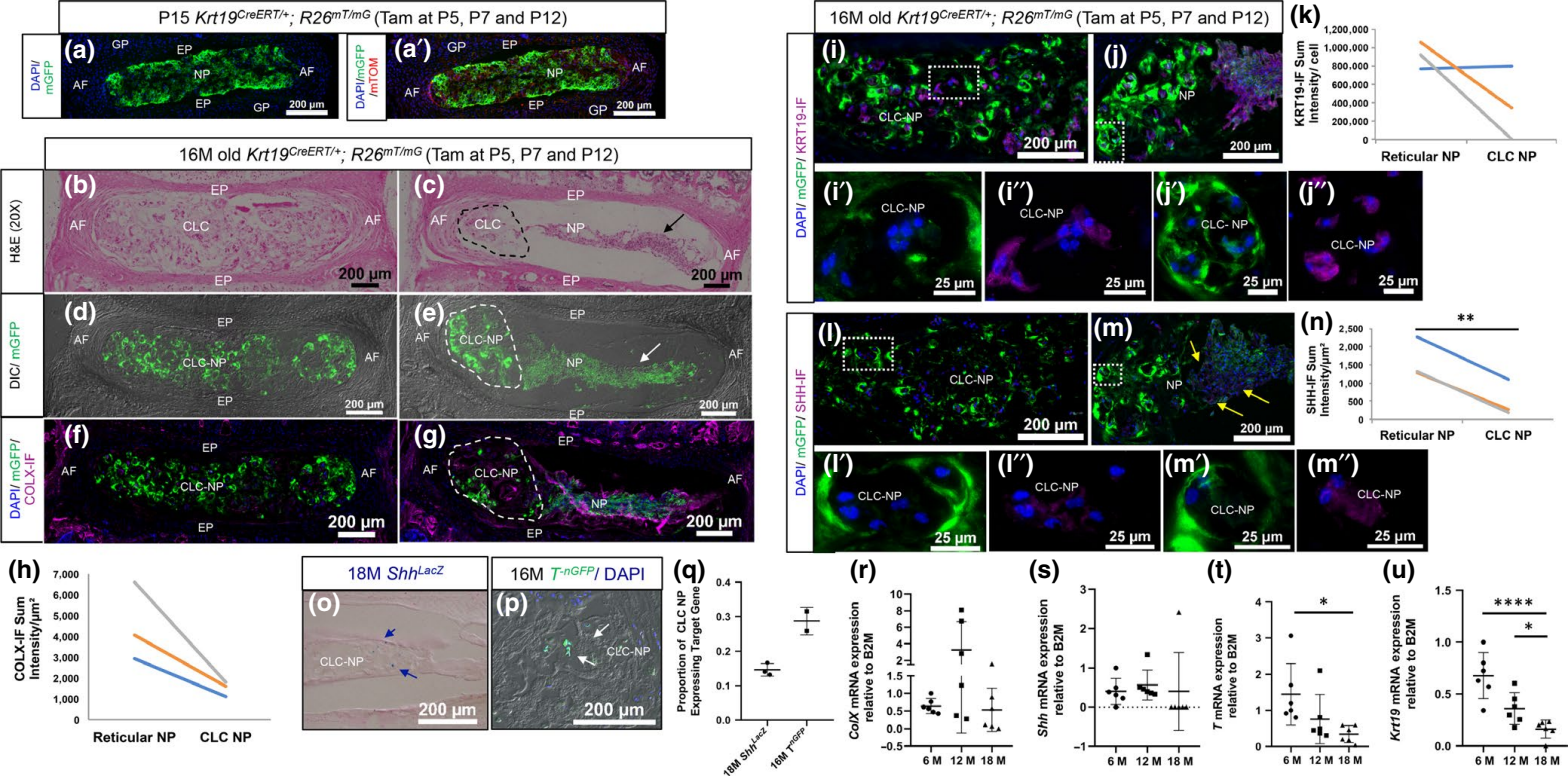
Fate mapping study reveals that CLC are late‐stage NP cells. a and a’, P15 Krt19^CreERT/+^; R26^mT/mG^ disc. b–m’’, coronal sections through two adjacent lumbar discs of fate‐mapped 16M old Krt19^CreERT/+^; R26^mT/mG^ mice. b and c, H&E staining, d and e, dark‐field (DIC) and epifluorescence for mGFP. f–m”, immunostaining (purple) and quantification of fluorescence intensity (*n* = 3) for; COLX (f–h); KRT19 (i–k); and SHH (l–n). Lines indicate comparison of CLC‐NP and reticular NP cells of adjacent discs from same mouse. o, β‐gal stained (blue cells, blue arrows) CLC‐NP in 18M old Shh^LacZ^ mice lumbar disc (*n* = 3). p, GFP+ (white arrows) CLC‐NP cells in lumbar disc from 16M T‐^nGFP^ mice (*n* = 2). qPCR for ColX (r), Shh (s), T (t) and Krt19 (u) using NP cells of 6M, 12M and 18M old mice (*n* = 6/age). P, postnatal day; M, month; GP, growth plate. *<.05, **<.01, ***<.001 ****<.0001 by paired t Test (h, k and n) or one‐way ANOVA (r, s, t and u)

To test the hypothesis that the NP differentiates into CLCs with aging, we crossed *Krt19^CreERT/+^* (Means, Xu, Zhao, Ray, & Gu, 2008) with *R26^mT/mG^* dual reporter (Muzumdar, Tasic, Miyamichi, Li, & Luo, 2007). In *Krt19^CreERT/+^*; *R26^mT/mG^* line*,* every cell initially expresses membrane‐bound tomato (mTOM+) and following tamoxifen treatment at P5, the mTOM cassette is removed and membrane‐bound EGFP (mGFP+) expression is turned “on” in the *Krt19*‐driven NP cells as seen at P15 (Figure 2a and a’). Some littermates were aged till 16M, and one of the lumbar discs was filled with CLCs (Figure 2b). These CLCs were mGFP+ and descendant of *Krt19*‐expressing NP cells (Figure 2d), indicating that NP cells differentiate into CLCs with aging. An adjacent disc from the same mouse displayed two phenotypes of mGFP+ cells that were spatially segregated: (a) compact but reticular NP (arrows) and (b) CLC‐NP (outlined, Figure 2c and e), suggesting that the differentiation of reticular NP into CLC‐NP occurs at different rates within the same spine. To determine whether CLC‐NP cells were similar to the hypertrophic chondrocytes of the growth plate, we performed immunostaining for type X collagen (COLX), a marker of hypertrophic chondrocytes (Kielty, Kwan, Holmes, Schor, & Grant, 1985) (Figure 2f and g). Quantification of immunofluorescence (IF) intensity shows reduced expression of COLX by CLC‐NP compared with reticular NP cells from adjacent discs of the same mouse (Figure 2h), indicating that the transition of NP cells into a CLC phenotype is not a hypertrophic stage of these cells, rather a pathological and terminal state.

Subsets of CLCs in aged human discs express KRT19 (Weiler et al., 2010). We tested the expression of KRT19 by immunostaining in the lineage‐traced CLC‐NPs and found that all CLCs and reticular NP cells were KRT19‐IF+, although KRT19 expression declined with differentiation (Figure 2i–k). We also observed that the nested cells in the lacunae of the CLC‐filled disc lacked individual cell membranes seen by an absence of mGFP+ signal around each cell at higher magnification (Figure 2i’ and l’). Instead, the differentiated CLCs resembled a syncytium, with one, intense mGFP+ signal emanating from a lacuna encasing several nuclei. In contrast, nested cells in the adjacent phenotypically mixed disc continued to maintain thinner cell membranes, although intense mGFP+ signal was observed at the surface of each lacuna (Figure 2j’ and m’). These observations suggest that the unidentified pericellular structure described by Trout et al. (1982) around the nested CLCs is generated by the fusion of cell membranes of several NP cells as they form a syncytia. Next, we analysed whether the differentiated NP cells continued to express SHH, which is crucial for proliferation and maintenance of the reticular structure of NP cells in the neonatal mouse lumbar disc (Dahia, Mahoney, & Wylie, 2012) and sacral disc (Bonavita, Vincent, Pinelli, & Dahia, 2018). *Shh* expression is known to decline from postnatal day four to one year of age in mouse NP cells (Dahia, Mahoney, Durrani, & Wylie, 2009; Winkler et al., 2014). Here, we observed that SHH‐IF decreased significantly (*p* < .01) in CLC‐NP compared with reticular NP cells (arrows) of adjacent disc from same mouse (Figure 2l–n). Moreover, SHH‐IF was confined within the lacunae and was detected only in a subset of lacunae. Next, to determine the number of CLC‐NP that express *Shh*, β‐gal staining was performed on the lumbar disc of 18M old *Shh^LacZ^* reporter mice, where *LacZ* is expressed under the *Shh* promoter and hence the *Shh*‐expressing cells can be analysed in real‐time (Gonzalez‐Reyes et al., 2012). In 18M old *Shh^LacZ^* mouse disc, 14.9% of CLC‐NP cells were β‐gal+ (Figure 2o and q). Results from previous studies show that SHH regulates expression of Brachyury (*Bra/T*) (Bonavita et al., 2018; Dahia et al., 2012), a key developmental molecule; hence, we analysed the expression of *Bra*, in CLC‐NP using its reporter allele where nuclear GFP is expressed under *Bra* promoter but inserted after the coding sequence of *Bra* gene (*T^‐nGFP^*, [Imuta, Kiyonari, Jang, Behringer, & Sasaki, 2013]). 28.8% of CLC‐NP cells were TnGFP+ in 16M old mouse lumbar discs (Figure 2p–q). These observations were validated by qPCR analysis. NP cells were dissected and pooled from all thoracic and lumbar discs of mice of different ages (Table S5) and RNA was isolated. Results confirmed decline in expression of *ColX*, *Shh, T and Krt19* in mouse NP cells with age (Figure 2r–u). However, as the NP cells were pooled from various discs and may represent NP cells with different phenotypes, qPCR results only show changes in gene expression associated with age, and not with NP phenotype. The results indicate that, although a subset of NP cells continue to express some of its unique molecular markers as they trans‐differentiate into a CLC phenotype, the expression of these markers is dramatically reduced, and expression of key signals including SHH was restricted within the lacunae.

Overall, these observations indicate that NP cells trans‐differentiate into smaller cells that fuse together to form a nest or syncytium with aging. Furthermore, this differentiation is associated with a decline in expression of key developmental molecules *Shh* and *Bra*. However, the precise mechanism of this differentiation remains uncertain. Previously, blockade of SHH signalling and its conditional targeting in neonatal mouse lumbar discs resulted in loss of BRA expression and change in NP cell phenotype from reticular to small round cells (Dahia et al., 2012). Recently, we observed that SHH and BRA expression reduced more rapidly and by 12 weeks of age in the cranial sacral discs of mouse spines. This, in turn, was associated with a change in NP cell phenotype from reticular‐shaped cells to collapsed and rounder cells (Bonavita et al., 2018). Further, conditional activation of hedgehog (Hh) signalling in a subset of NP cells of 12‐week‐old mice increased BRA expression in all NP cells and reversed the phenotype of NP cells from small and round back to reticular, indicating that in the sacral discs differentiation of NP cells is regulated by SHH signalling. It is likely that decline in SHH expression with physiological aging in the lumbar disc, and restriction of signalling potential within a subset of lacunae, results in loss of Hh response, which drives the differentiation of NP cells into a CLC like phenotype. It is also likely that elevated levels of inflammatory cytokines during normal aging and disc degeneration (reviewed by [Risbud & Shapiro, 2014]) play a role in differentiation of NP cells into CLCs. Future studies aimed at elucidating the molecular mechanism regulating progression of healthy and larger NP cells into their terminally differentiated state will provide a platform for research aimed at preventing disc aging and curing back pain.

## CONFLICT OF INTEREST

None declared.

## AUTHOR CONTRIBUTIONS

CLD conceived and designed the study, interpreted the data and supervised the project. SM, RP, PP and CLD carried out the experiments and generated the data. CLD, SM, PP and TJA analysed the data. CLD and SM wrote the manuscript. All authors reviewed the manuscript and gave their final approval for submission.
