# Supplementary material for: Chondrocyte‐like nested cells in the aged intervertebral disc are late‐stage nucleus pulposus cells
Source: Aging Cell. 2019 Jul 10;18(5):e13006. doi: 10.1111/acel.13006 (PMC6718620; doi:10.1111/acel.13006)
Supplement: Supplementary file 1 [file ACEL-18-e13006-s001.pdf]

SUPPLEMENTAL DATA:

Experimental procedures:

Mice

Male and female mice of different ages and genetic background were used. All mice were maintained in accordance with National Institutes of Health Guide for the Care and Use of Laboratory Animals, and all experiments were carried out in accordance with institutional guidelines under Weill Cornell Medical College (WCMC) Institutional Animal Care and Use Committee (IACUC). Mice were housed under 12 hours of light/ dark cycle in the WCMC animal facility with food and water *ad libitum*.

Histochemical Staining and Histopathological analysis

The lumbar spines were dissected from mice of given age into ice-cold phosphate buffered saline (PBS) and immediately fixed in 4% paraformaldehyde (PFA) for four hours. Decalcification of the adult mouse spine was carried out using 0.5 M ethylenediaminetetraacetic acid (EDTA, Sigma-Aldrich, USA, E9884), pH 7.6 at 8°C on a rocker platform for nine days. Following decalcification, the spines were washed three times in cold PBS for 30 min each. Spines were prepared for cryosectioning in Tissue-Tek® optimum cutting temperature (O.C.T, VWR, USA, 102094-106) molds, snap frozen, and stored at -80°C until further use. Cryosections in the coronal plane were prepared at 8 µm thickness using a Leica cryostat. Slides were stored at -80°C. Mid-coronal sections were processed for histological evaluation by hematoxylin and eosin (H&E) staining using a standard protocol, dehydrated, cleared and mounted in PROTOCOL™ mounting medium (Fisher Healthcare™, USA, C.A.S. 23-245-691).

For Safranin-O (SafO) and Fast Green Staining, frozen sections were hydrated in distilled water for 5 min and then incubated for 12 min in aqueous 0.001% Fast Green Solution (Sigma-Aldrich, USA, 42053). Fast green stained sections were de-stained by rinsing with 1% acetic acid solution and subsequently stained with 0.1% Safranin-O solution (Sigma-Aldrich, USA, 50240) for 20 min. The sections were then dehydrated, cleared, and mounted in PROTOCOL™ mounting medium.

For Picrosirius red staining, frozen sections were hydrated in distilled water for 5 min and incubated for 30 min with 0.1% Picrosirius Red staining Solution prepared by dissolving Sirius red (Polysciences, USA, 09400) in saturated aqueous picric acid (Sigma-Aldrich, USA, P6744). Sections were de-stained by rinsing with 0.01% hydrochloric acid solution for 10 seconds and then dehydrated, cleared and mounted in PROTOCOL^{TM} mounting medium.

The histopathological changes in the lumbar IVDs with age were quantified using scoring criteria published by Tam et al., 2017 (Tam et al., 2018). Briefly, H&E and SafO/ Fast green stained mid-coronal serial sections from lumbar spines of mice aged between 12 to 28 months of age were scored independently by two blind reviewers. The details on cohorts are provided in Table S1. L3 to S1 discs were scored using the five criteria (Tam et al., 2018). For NP structure (0 – 4), NP cleft/ fissures (0 – 2), AF structure (0 – 4), AF cleft/ fissures (0 – 2), NP/AF boundary (0 – 2), where the increase in score indicates pathological changes in each category. “NP” score for each disc was calculated by adding the score for the NP structure and NP cleft/ fissures. “AF” score was calculated by adding the score for the AF structure and NP cleft/ fissures. Total score for each disc was determined by adding all five scores for that disc. Averages were calculated for each age range and standard error of mean (SEM) was calculated. Increase in total pathological score across L3 - L4 through L6 - S1 were reported in Table S2 and Figure 1m. As the changes were more dramatic between L5 – S1 discs, increase in pathology of NP, AF, and NP/AF boundary at the L5 - L6 and L6 - S1 levels were analyzed in Table S3 and Figure 1n. Scores for disc histopathology was analyzed by one-way ANOVA at four levels (L3 - L4, L4 - L5, L5 - L6, L6 - S1) with the relevant factor being the age of the sample (12 -14M, 15 -18M, 19 to 21M, and 22-28M). A significant main effect of either factor prompted Tukey post-hoc analyses for multiple comparisons. A p-value of less than 0.05 was considered statistically significant.

Lumbar intervertebral discs were imaged at 10x or 60x magnification using a Nikon Eclipse wide-field microscope and accompanying NIS Elements AR software (Nikon, Japan).

Fate-mapping Studies

Recently we showed that the tamoxifen inducible *Krt19*$^{CreERT/+}$ (Means et al., 2008) allele specifically and efficiently mediates recombination in NP cells in the spine (Mohanty et al., 2019). For fate-mapping NP cells *Krt19*$^{CreERT/+}$ was crossed with *Gt(ROSA)26Sor*$^{tm4(ACTB-tdTomato,-EGFP)Luo}$/J [*R26*$^{mT/mG}$, (Muzumdar et al., 2007)] double fluorescent conditional reporter to generate the *Krt19*$^{CreERT/+}$; *R26*$^{mT/mG}$ line (Mohanty et al., 2019). Mice were genotyped using specific primers for each transgene as previously described (Means et al., 2008; Muzumdar et al., 2007). Tamoxifen (Sigma-Aldrich, USA T5648) was dissolved to 20 mg/ml concentration in corn oil by incubating in a 37°C shaker overnight. Tamoxifen was administered intraperitoneally at P5, P7 and P12 at a dose of 200 µg/gm body weight (Mohanty et al., 2019). The lumbar spine was collected at the time of euthanasia, fixed, and processed for cryosectioning as described above under Histochemical Staining section. To determine recombination, the slides were washed three times in PBS, counter-stained in DAPI (1:5000, Life Technologies, USA, D1306), and mounted in Prolong™ Diamond (Life Technologies, USA, P36962) and imaged using a Nikon Eclipse Epi-Fluorescence microscope and accompanying NIS Elements AR software (Nikon, Japan).

Immunofluorescence Analysis

Immunostaining for lumbar intervertebral discs was carried out as previously described (Dahia et al., 2009). At least three biological replicates having one lumbar disc with CLC-NP and an adjacent disc with mostly reticular-NP cells were processed. Briefly, mid-coronal cryosections of lumbar spines were air-dried and washed twice in PBS for 5 min each. Sections were permeabilized in PBS containing 0.25% Triton-x 100 (0.25% PBST) for 20 mins. The sections were blocked in blocking buffer [10% Donkey serum (Jackson ImmunoResearch, USA, 017-000-121), 4% IgG-free BSA (Jackson ImmunoResearch, USA, 001-000-162) and 0.1% PBST] for one hour at room temperature in a humidified chamber. Sections were hybridized with specific primary antibodies [KRT19/ TROMAIII, MM IgG (1:100) (DSHB, USA, TROMA-III); SHH, RatM IgG (1:50) (Sigma USA, S4944); COLX, RP IgG (1:100) (abcam USA, ab58632) (all dilutions were prepared in blocking buffer)] at 4°C overnight in a humidified chamber.

Slides were then washed three times in PBS and hybridized with Alexa Fluor® 647 conjugated secondary antibodies, Alexa Fluor® 647-AffiniPure Goat Anti-Mouse IgG (115-605-146); Alexa Fluor® 647-AffiniPure Goat Anti-Rat IgG (112-605-062); Alexa Fluor® 647-AffiniPure Donkey Anti-Rabbit IgG (711-605-144), (all from Jackson ImmunoResearch, USA), at a 1:200 dilution in blocking buffer and incubated in the dark for one hour at room temperature in a humidified chamber. Next, the slides were washed three times in PBS, counterstained with DAPI (1:5000, Life Technologies, USA, D1306), and mounted in ProLong™ Gold (Life Technologies, USA, P36934). Control slides were incubated only with the appropriate secondary antibody. All controls showed negative staining (data not shown). Serial sections were imaged using a Nikon Eclipse Epi-Fluorescence microscope and NIS Elements AR software.

Quantification of Immunofluorescence Intensity

Quantification of immunofluorescence intensity was performed using the Nikon NIS-AR Elements software (Nikon, Japan) as described previously (Bonavita et al., 2018). To calculate the total fluorescence intensity per reticular-NP cell for KRT19 immunofluorescence, 100 individual regions of interest, corresponding to 100 individual NP cells per disc, were selected. The cell membrane was determined by using the mGFP and mTOM natural fluorescence along with the dark-field using differential interference contrast (DIC) objective. “ROI Sum Intensities” of each cell, which correspond to the total fluorescent intensity per cell, was averaged. For CLC-NP cells, the surface of the lacunae, which corresponded to an intense mGFP signal, was selected to determine the boundary of the ROI. The number of nuclei per lacunae were counted manually until 50 to 100 total CLC-NP were analyzed per disc. The “ROI sum intensity” was summed across all CLC regions of interest and divided by number of cells observed to attain the fluorescent intensity per CLC-NP cell.

To assess levels of SHH and COLX in reticular NP cells, the ROI selected corresponded to a region occupied ubiquitously and solely by NP cells. Here, the “ROI sum intensity” was divided by the “ROI area” to attain the metric: total fluorescence intensity per um^2. For CLC-NP, each ROI corresponded to single lacunae. Similarly, the “ROI sum intensities” were summed across all lacunae present within an individual IVD

and subsequently divided by the sum of the ROI areas. Adjacent IVDs from same mouse spine where one disc consisted of reticular-NP and the other consisted predominantly of CLC-NP cells were analyzed for KRT19, SHH, and COLX protein expression by immunostaining. The difference in IF intensity between reticular NP and CLC-NP of adjacent discs in three biological samples is indicated by line in Fig. 2. Subsequently, two-tailed, paired t-tests were conducted. A p-value of less than 0.05 was considered statistically significant.

Quantification for Cell counts

To assess how the number and phenotype of NP cells changes with age in Figure 1o and Table S4, a region of interest (ROI) was defined around the NP cells. At least three serial sections were used as technical replicates for each biological sample. For samples counterstained with DAPI, the total number of nuclei within the ROI were counted by thresholding for DAPI using the NIS software on serial sections. The phenotype (reticular or CLC) of the NP cells was assessed manually and the proportion of reticular NP or CLC-NP was calculated for four cohorts (12 – 14M, 15 – 18M, 19 – 21M, 22 -28M). The CLC-NP cells were defined as the cells being encased in lacunae. The data was analyzed by one-way ANOVA at one level (L5 - L6) with the relevant factor being the age of the sample (12 -14M, 15 -18M, 19 - 21M, and 22 - 28M). One-way ANOVA was conducted independently for the parameters: total NP cells, proportion of reticular NP, and proportion of CLC-NP. A significant main effect of either factor prompted Tukey post-hoc analyses for multiple comparisons. A p-value of less than 0.05 was considered statistically significant.

To assess the proportion of CLC-NP cells expressing *Shh*, the total nuclei and the proportion of Shh^{LacZ}+ (blue) cells were counted manually in IVDs of 18M old Shh^{LacZ} mice (Gonzalez-Reyes et al., 2012).

To assess the proportion of CLC-NP cells expressing *T*, the total number of nuclei were counted by thresholding for DAPI, and DIC filters using the NIS software described above. The nuclei of $T\text{-}^{nGFP}$+ CLCs were counted manually in 16M old $T\text{-}^{nGFP}$ mice (Imuta et al., 2013). Mean ± SEM is reported.

RNA isolation and qPCR analysis

For mRNA expression analysis, NP cells were collected from at least three males and three females for each cohort of six, 12, and 18 months of age (Table S5). NP cells were micro-dissected in cold PBS under a Nikon stereomicroscope (Nikon, Japan). All NP cells micro-dissected from thoracic and lumbar IVDs of each mouse were pooled in Eppendorf tube containing 500 µl of RNA*later*™ (Invitrogen by Thermo Fisher Scientific, Lithuania, AM7024) and incubated at 4°C for 24 hrs. The next day, the RNA*later*™ was removed and NP cells were stored at -80°C until further use. RNA was isolated from NP cells by homogenizing using the Polytron Omni Tissue Homogenizer (Omni International, USA, LR60902) in using one ml of Tri-Reagent (Sigma-Aldrich, USA, 93289). The homogenate was centrifuged at 12,000 rpm at 4°C for 12 min and the supernatant was collected in a fresh Eppendorf tube and incubated at room temperature for 10 mins. Next, $1/5^{th}$ volume of chloroform (Sigma-Aldrich, USA, C2432) was added to each sample, mixed, and samples were further incubated for 10 min at room temperature, following which the sample was centrifuged at 12,000 rpm for 15 min and at 4°C. The top aqueous phase was pipetted out into a new tube to which equal volume of RNase-free 70% alcohol was added and mixed. The samples were then transferred to Qiagen RNeasy Mini Spin Columns (Qiagen, Germany, 74104) and RNA was isolated per manufacturer's protocol. Following elution, the RNA concentration was quantified in duplicates using NanoDrop™ One Microvolume UV-Vis Spectrophotometer (Thermo Scientific, USA, AZY1601393). This protocol is standardized for isolating high quality RNA with RNA integrity score (RIN) of 9 and above as assessed using BioAnalyzer. Following isolation the RNA was immediately converted into cDNA using SuperScript™ IV First-Strand Synthesis System (Invitrogen by Thermo Fisher Scientific, Lithuania, 18091050). Expression of *ColX* (Thermo Fisher Scientific, USA, Mm00443177_m1), *Krt19* (Thermo Fisher Scientific, USA, Mm00492980_m1), *Shh* (Thermo Fisher Scientific, USA, Mm00436528_m1)*,* and *T* (Thermo Fisher Scientific, USA, Mm01318249_m1) was analyzed using 6ng of cDNA and iQ™ Multiplex Powermix (Bio-Rad, USA, 1725849) master mix using gene specific TaqMan probes conjugated to FAM, multiplexed with Beta-2 Microglobulin (*B2m*, Thermo Fisher Scientific, USA, Mm00437762_m1) conjugated to VIC as internal control for every

assay, and using CFX96 Touch™ Real-Time PCR Detection System(Bio-Rad, Singapore, 1855195). The delta-delta CT (DDCT) was calculated and log-2 fold change was determined relative to 6-month-old NP cells. Data is presented by calculating the averages and presented as Mean ± SEM. Changes in gene expression with age was analyzed by one-way ANOVA at three ages (6M, 12M, and 18M). A significant main effect of either factor prompted Tukey post-hoc analyses for multiple comparisons. A p-value of less than 0.05 was considered statistically significant.

Beta-Galactosidase Staining

Cryosections of lumbar spine from 18M old *Shh*^{LacZ} mice were washed twice for 10 min each in wash buffer (2 mM $MgCl_2$, Sigma-Aldrich, USA, M8266), and were permeabilized using 0.2% IGEPAL® CA-630 (Sigma-Aldrich, USA, I8896) and 2 mM $MgCl_2$. Sections were incubated in β-galactosidase staining solution [35 mM $K_3Fe(CN)_6$ (Sigma-Aldrich, USA, 244023), 35 mM $K_4Fe(CN)_6$ (Sigma-Aldrich, USA, P3289), 2 mM $MgCl_2$, 0.01% NaDeoxycholate (Sigma-Aldrich, USA, D6750), 0.02% IGEPAL® CA-630, 1 mg/ml β-galactosidase (Sigma-Aldrich, USA, B4252), 0.5 M EGTA (Sigma-Aldrich, USA , E3889) in PBS] for four hours at 37°C in a humidified chamber. After washing twice in wash buffer, the sections were counterstained with nuclear fast red, dehydrated in an increasing gradient of ethanol, cleared in xylene, and mounted using xylene based mounting medium. The nested chondrocyte-like NP cells were imaged using DIC filters on the Nikon Eclipse microscope.

Statistical analysis

Statistical analysis was performed using GraphPad Prism version 8.0.

Supplemental Figure S1

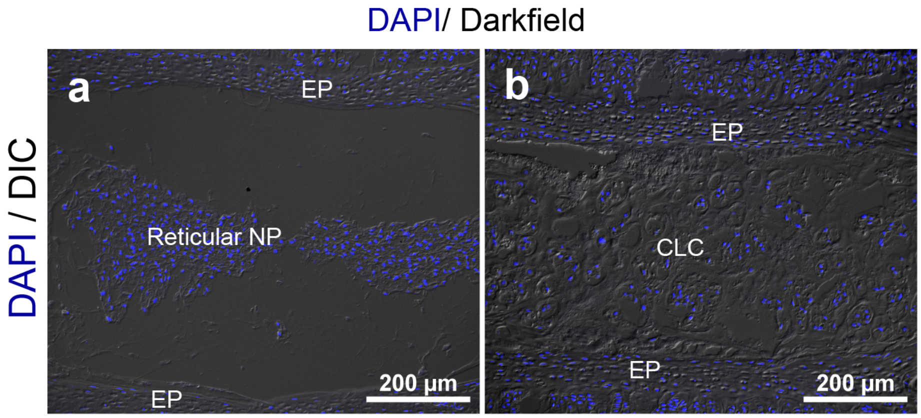

Figure S1. Quantification of Reticular NP and CLCs in lumbar disc with age.

Representative images from 16M *Krt19*$^{CreERT/+}$*; R26*$^{mT/mG}$ mouse lumbar spine showing a coronal view of disc with reticular NP cells (**a**) and an adjacent disc from the same spine with CLCs (**b**) imaged using dark-field (DIC) to distinguish the two phenotypes. Nuclei are counterstained with DAPI (*blue*). The NP cells were counted using DAPI filter.

Supplemental Table S1. Demographics of Animals Used for Histopathological Scoring of intervertebral discs

Age	n	# of Female(Male)	% of Female(Male)
12 – 14M	15	5(10)	33.3(66.7)
15 – 18M	12	7(5)	58.3(41.7)
19 – 21M	9	2(7)	22.2(79.8)
22 – 28M	20	3(17)	15.0(85.0)

Supplemental Table S2. Comparison of Histopathological Scores

a. Comparison of Total Pathological Score from L3 - S1 at Different Ages.				
**Tukey's multiple comparisons test**	**Mean Diff.**	**95.00% CI of diff.**	**Summary**	**Adjusted P Value**
12 - 14M vs. 15 - 18M	-2.404	-6.866 to 2.058	ns	0.4609
12 - 14M vs. 19 - 21M	-11.21	-15.93 to -6.491	****	<0.0001
12 - 14M vs. 22 - 28M	-10.43	-14.72 to -6.144	****	<0.0001
15 - 18M vs. 19 - 21M	-8.808	-12.03 to -5.591	****	<0.0001
15 - 18M vs. 22 - 28M	-8.026	-10.56 to -5.490	****	<0.0001
19 - 21M vs. 22 - 28M	0.7821	-2.188 to 3.752	ns	0.8855
b. Comparison of L3 - L4 Total Pathological Score (One-way ANOVA)				
**Tukey's multiple comparisons test**	**Mean Diff.**	**95.00% CI of diff.**	**Summary**	**Adjusted P Value**
12 - 14M vs. 15 - 18M	1.67	-3.879 to 7.219	ns	0.8408
12 - 14M vs. 19 - 21M	-1.5	-7.439 to 4.439	ns	0.8981
12 - 14M vs. 22 - 28M	0	-5.392 to 5.392	ns	>0.9999

15 - 18M vs. 19 - 21M	-3.17	-7.129 to 0.7894	ns	0.1502
15 - 18M vs. 22 - 28M	-1.67	-4.748 to 1.408	ns	0.4569
19 - 21M vs. 22 - 28M	1.5	-2.236 to 5.236	ns	0.6901
c. Comparison of L4 - L5 Total Pathological Score (One-way ANOVA)				
**Tukey's multiple comparisons test**	**Mean Diff.**	**95.00% CI of diff.**	**Summary**	**Adjusted P Value**
12 - 14M vs. 15 - 18M	0.27	-3.120 to 3.660	ns	0.9965
12 - 14M vs. 19 - 21M	-0.63	-4.255 to 2.995	ns	0.9659
12 - 14M vs. 22 - 28M	-1.53	-4.466 to 1.406	ns	0.508
15 - 18M vs. 19 - 21M	-0.9	-4.525 to 2.725	ns	0.9091
15 - 18M vs. 22 - 28M	-1.8	-4.736 to 1.136	ns	0.3661
19 - 21M vs. 22 - 28M	-0.9	-4.104 to 2.304	ns	0.8745
d. Comparison of L5 - L6 Total Pathological Score (One-way ANOVA)				
**Tukey's multiple comparisons test**	**Mean Diff.**	**95.00% CI of diff.**	**Summary**	**Adjusted P Value**
12 - 14M vs. 15 - 18M	-1.682	-5.275 to 1.911	ns	0.6014
12 - 14M vs. 19 - 21M	-3.214	-7.156 to 0.7271	ns	0.1462
12 - 14M vs. 22 - 28M	-3.35	-6.475 to -0.2252	*	0.0313
15 - 18M vs. 19 - 21M	-1.532	-5.608 to 2.543	ns	0.7497
15 - 18M vs. 22 - 28M	-1.668	-4.961 to 1.624	ns	0.5373
19 - 21M vs. 22 - 28M	-0.1357	-3.805 to 3.533	ns	0.9997
e. Comparison of L6 - S1 Total Pathological Score (One-way ANOVA)				
**Tukey's multiple comparisons test**	**Mean Diff.**	**95.00% CI of diff.**	**Summary**	**Adjusted P Value**

12 - 14M vs. 15 - 18M	-2.952	-6.913 to 1.009	ns	0.2063
12 - 14M vs. 19 – 21M	-5.91	-10.04 to -1.778	**	0.0023
12 - 14M vs. 22 - 28M	-6.018	-9.265 to -2.771	****	<0.0001
15 - 18M vs. 19 – 21M	-2.958	-7.520 to 1.603	ns	0.3185
15 - 18M vs. 22 - 28M	-3.066	-6.845 to 0.7128	ns	0.148
19 - 21M vs. 22 - 28M	-0.1078	-4.066 to 3.850	ns	0.9999

Supplemental Table S3. Comparison of L5 – L6 and L6 – S1 Pathological Scores

a. Comparison of L5 - L6 NP Pathological Score (One-way ANOVA)				
**Tukey's multiple comparisons test**	**Mean Diff.**	**95.00% CI of diff.**	**Summary**	**Adjusted P Value**
12 - 14M vs. 15 - 18M	-1.021	-2.896 to 0.8540	ns	0.4752
12 - 14M vs. 19 – 21M	-1.67	-3.816 to 0.4753	ns	0.1768
12 - 14M vs. 22 - 28M	-1.66	-3.290 to -0.02907	*	0.0446
15 - 18M vs. 19 – 21M	-0.6494	-2.862 to 1.564	ns	0.8624
15 - 18M vs. 22 - 28M	-0.6386	-2.357 to 1.079	ns	0.7558
19 - 21M vs. 22 - 28M	0.01071	-1.999 to 2.021	ns	>0.9999
b. Comparison of L5 - L6 AF Pathological Score (One-way ANOVA)				
**Tukey's multiple comparisons test**	**Mean Diff.**	**95.00% CI of diff.**	**Summary**	**Adjusted P Value**
12 - 14M vs. 15 - 18M	-0.1853	-1.353 to 0.9824	ns	0.9743
12 - 14M vs. 19 – 21M	-0.8736	-2.210 to 0.4626	ns	0.3144
12 - 14M vs. 22 - 28M	-0.8558	-1.871 to 0.1597	ns	0.1262
15 - 18M vs. 19 – 21M	-0.6883	-2.066 to 0.6898	ns	0.5487
15 - 18M vs. 22 - 28M	-0.6705	-1.740 to 0.3995	ns	0.3513

19 - 21M vs. 22 - 28M	0.01786	-1.234 to 1.270	ns	>0.9999
c. Comparison of Pathological Score for L5 - L6 NP/AF Boundary (One-way ANOVA)				
**Tukey's multiple comparisons test**	**Mean Diff.**	**95.00% CI of diff.**	**Summary**	**Adjusted P Value**
12 - 14M vs. 15 - 18M	-0.4755	-1.220 to 0.2687	ns	0.3342
12 - 14M vs. 19 - 22M	-0.6703	-1.522 to 0.1813	ns	0.1692
12 - 14M vs. 22 - 28M	-0.8346	-1.482 to -0.1874	**	0.0066
15 - 18M vs. 19 – 21M	-0.1948	-1.073 to 0.6835	ns	0.9344
15 - 18M vs. 22 - 28M	-0.3591	-1.041 to 0.3228	ns	0.5041
19 - 21M vs. 22 - 28M	-0.1643	-0.9620 to 0.6335	ns	0.9465
d. Comparison of L6 - S1 NP Pathological Score (One-way ANOVA)				
**Tukey's multiple comparisons test**	**Mean Diff.**	**95.00% CI of diff.**	**Summary**	**Adjusted P Value**
12 - 14M vs. 15 - 18M	-1.673	-3.712 to 0.3657	ns	0.1408
12 - 14M vs. 19 – 21M	-2.84	-5.079 to -0.6005	**	0.0081
12 - 14M vs. 22 - 28M	-3.1	-4.771 to -1.428	****	<0.0001
15 - 18M vs. 19 – 21M	-1.167	-3.617 to 1.284	ns	0.5831
15 - 18M vs. 22 - 28M	-1.426	-3.372 to 0.5188	ns	0.218
19 - 21M vs. 22 - 28M	-0.2598	-2.414 to 1.895	ns	0.9882
e. Comparison of L6 - S1 AF Pathological Score (One-way ANOVA)				
**Tukey's multiple comparisons test**	**Mean Diff.**	**95.00% CI of diff.**	**Summary**	**Adjusted P Value**
12 - 14M vs. 15 - 18M	-0.7837	-2.223 to 0.6561	ns	0.4732

12 - 14M vs. 19 - 21M	-1.763	-3.344 to -0.1815	*	0.0236
12 - 14M vs. 22 - 28M	-1.758	-2.899 to -0.6165	***	0.001
15 - 18M vs. 19 – 21M	-0.9792	-2.709 to 0.7512	ns	0.4392
15 - 18M vs. 22 - 28M	-0.9743	-2.315 to 0.3660	ns	0.2259
19 - 21M vs. 22 - 28M	0.004902	-1.486 to 1.496	ns	>0.9999
f. Comparison of Pathological Score for L6 - S1 NP/AF Boundary (One-way ANOVA)				
**Tukey's multiple comparisons test**	**Mean Diff.**	**95.00% CI of diff.**	**Summary**	**Adjusted P Value**
12 - 14M vs. 15 - 18M	-0.4952	-1.283 to 0.2928	ns	0.3451
12 - 14M vs. 19 – 21M	-1.308	-2.173 to -0.4422	**	0.0013
12 - 14M vs. 22 - 28M	-1.161	-1.807 to -0.5145	***	0.0001
15 - 18M vs. 19 – 21M	-0.8125	-1.760 to 0.1345	ns	0.1152
15 - 18M vs. 22 - 28M	-0.6654	-1.417 to 0.08641	ns	0.099
19 - 21M vs. 22 - 28M	0.1471	-0.6856 to 0.9798	ns	0.9645

g. Comparison of L5 - L6 vs. L6 - S1 for All Parameters by Two Tailed T-Test				
**Age Cohort**	**NP**	**AF**	**NP/ AF Boundary**	**Total Score(NP, AF, Boundary)**
12 - 14M	0.886	0.6353	0.5743	0.8834
15 - 18M	0.463	0.3731	0.728	0.424
19 - 21M	0.368	0.3309	0.193	0.2662
22 - 28M	0.04	0.062	0.1224	0.0471

Supplemental Table S4. Appearance of CLC NP with Age

a. Comparison of Total Number of NP Cells with Age				
**Tukey's multiple**	**Mean Diff.**	**95.00% CI of diff.**	**Summary**	**Adjusted**

comparisons test				P Value
12 - 14M vs. 15 - 18M	345.2	246.0 to 444.4	****	<0.0001
12 - 14M vs. 19 - 21M	374.3	275.2 to 473.5	****	<0.0001
12 - 14M vs. 22 - 28M	433.8	342.0 to 525.6	****	<0.0001
15 - 18M vs. 19 - 21M	29.11	-76.90 to 135.1	ns	0.8344
15 - 18M vs. 22 - 28M	88.57	-10.59 to 187.7	ns	0.0838
19 - 21M vs. 22 - 28M	59.46	-39.70 to 158.6	ns	0.3135
b. Decreasing Proportion of Reticular NP Cells with Age				
**Tukey's multiple comparisons test**	**Mean Diff.**	**95.00% CI of diff.**	**Summary**	**Adjusted P Value**
12 - 14M vs. 15 - 18M	0.7764	0.1323 to 1.420	*	0.0184
12 - 14M vs. 19 - 21M	0.6667	0.02261 to 1.311	*	0.0421
12 - 14M vs. 22 - 28M	1	0.4037 to 1.596	**	0.0021
15 - 18M vs. 19 - 21M	-0.1097	-0.7982 to 0.5788	ns	0.9602
15 - 18M vs. 22 - 28M	0.2236	-0.4204 to 0.8677	ns	0.7187
19 - 21M vs. 22 - 28M	0.3333	-0.3107 to 0.9774	ns	0.4292
c. Increasing Proportion of CLC NP Cells with Age				
**Tukey's multiple comparisons test**	**Mean Diff.**	**95.00% CI of diff.**	**Summary**	**Adjusted P Value**
12 - 14M vs. 15 - 18M	-0.7764	-1.420 to -0.1323	*	0.0184
12 - 14M vs. 19 - 21M	-0.6667	-1.311 to -0.02261	*	0.0421
12 - 14M vs. 22 - 28M	-1	-1.596 to -0.4037	**	0.0021
15 - 18M vs. 19 - 21M	0.1097	-0.5788 to 0.7982	ns	0.9602
15 - 18M vs. 22 - 28M	-0.2236	-0.8677 to 0.4204	ns	0.7187

19 - 21M vs. 22 - 28M	-0.3333	-0.9774 to 0.3107	ns	0.4292

Supplemental Table S5. Demographics of Animals Used for qPCR

Age	n	% of F(M)	# of F(M)
6M	6	50(50)	3(3)
12M	6	50(50)	3(3)
18M	6	50(50)	3(3)

References:

Bonavita, R., Vincent, K., Pinelli, R., & Dahia, C. L. (2018). Formation of the sacrum requires down-regulation of sonic hedgehog signaling in the sacral intervertebral discs. *Biol Open, 7*(7). doi: 10.1242/bio.035592

Gonzalez-Reyes, L. E., Verbitsky, M., Blesa, J., Jackson-Lewis, V., Paredes, D., Tillack, K., . . . Kottmann, A. H. (2012). Sonic hedgehog maintains cellular and neurochemical homeostasis in the adult nigrostriatal circuit. *Neuron, 75*(2), 306-319. doi: 10.1016/j.neuron.2012.05.018

Imuta, Y., Kiyonari, H., Jang, C. W., Behringer, R. R., & Sasaki, H. (2013). Generation of knock-in mice that express nuclear enhanced green fluorescent protein and tamoxifen-inducible Cre recombinase in the notochord from Foxa2 and T loci. *Genesis, 51*(3), 210-218. doi: 10.1002/dvg.22376

Means, A. L., Xu, Y., Zhao, A., Ray, K. C., & Gu, G. (2008). A CK19(CreERT) knockin mouse line allows for conditional DNA recombination in epithelial cells in multiple endodermal organs. *Genesis, 46*(6), 318-323. doi: 10.1002/dvg.20397

Mohanty, S., Pinelli, R., & Dahia, C. L. (2019). Characterization of Krt19 (CreERT) allele for targeting the nucleus pulposus cells in the postnatal mouse intervertebral disc. *J Cell Physiol*. doi: 10.1002/jcp.28952

Muzumdar, M. D., Tasic, B., Miyamichi, K., Li, L., & Luo, L. (2007). A global double-fluorescent Cre reporter mouse. *Genesis, 45*(9), 593-605. doi: 10.1002/dvg.20335

Tam, V., Chan, W. C. W., Leung, V. Y. L., Cheah, K. S. E., Cheung, K. M. C., Sakai, D., . . . Chan, D. (2018). Histological and reference system for the analysis of mouse intervertebral disc. *J Orthop Res, 36*(1), 233-243. doi: 10.1002/jor.23637
